# Enhancing Gen3 for clinical trial time series analytics and data discovery: a data commons framework for NIH clinical trials

**DOI:** 10.3389/fdgth.2025.1570009

**Published:** 2025-07-23

**Authors:** Meredith C. B. Adams, Colin Griffin, Hunter Adams, Stephen Bryant, Robert W. Hurley, Umit Topaloglu

**Affiliations:** ^1^ Department of Anesthesiology, Wake Forest University School of Medicine, Winston-Salem, NC, United States; ^2^ Division of Public Health Sciences, Wake Forest University School of Medicine, Winston-Salem, NC, United States; ^3^ Department of Translational Neuroscience, Wake Forest University School of Medicine, Winston-Salem, NC, United States; ^4^ Krumware LLC, Columbia, SC, United States; ^5^ Department of Cancer Biology, Wake Forest University School of Medicine, Winston-Salem, NC, United States; ^6^ Clinical Translational Research Informatics Branch, National Cancer Institute, National Institutes of Health, Rockville, MD, United States

**Keywords:** data commons, cloud computing, opioid, chronic pain, Kubernetes, Gen3, timeseries, patient reported outcomes

## Abstract

This work presents a framework for enhancing Gen3, an open-source data commons platform, with temporal visualization capabilities for clinical trial research. We describe the technical implementation of cloud-native architecture and integrated visualization tools that enable standardized analytics for longitudinal clinical trial data while adhering to FAIR principles. The enhancement includes Kubernetes-based container orchestration, Kibana-based temporal analytics, and automated ETL pipelines for data harmonization. Technical validation demonstrates reliable handling of varied time-based data structures, while maintaining temporal precision and measurement context. The framework's implementation in NIH HEAL Initiative networks studying chronic pain and substance use disorders showcases its utility for real-time monitoring of longitudinal outcomes across multiple trials. This adaptation provides a model for research networks seeking to enhance their data commons capabilities while ensuring findable, accessible, interoperable, and reusable clinical trial data.

## Introduction

Clinical trial networks require sophisticated data commons platforms that support longitudinal analytics while following FAIR (Findable, Accessible, Interoperable, and Reusable) data principles (1). While existing data commons solutions excel at managing static datasets, they often lack native capabilities for tracking and visualizing the progression of clinical outcomes over time (2, 3). This gap presents a significant challenge for understanding treatment effectiveness in interventional studies.

Current data commons platforms face several technical limitations when applied to longitudinal clinical research. These specific technical requirements include: the need for temporal data modeling that preserves measurement timing and sequence, standardized harmonization of patient-reported outcome measures across multiple trials, real-time monitoring capabilities for trial management, and integration of diverse data types with varying collection frequencies from daily patient reports to monthly clinical assessments. Existing platforms typically require specialized informatics support and custom development for temporal analyses, limiting accessibility for clinical researchers.

Gen3, an open-source platform widely adopted across NIH-funded research networks, provides robust capabilities for data storage, access control, and basic querying (4). However, its implementation for clinical trial visualization has revealed important capability gaps, particularly in temporal analysis and standardized outcome measure harmonization (5). The complexity increases with requirements for integrating diverse data types from multiple concurrent trials, implementing granular access controls for multi-site studies, and supporting real-time monitoring of recruitment and outcomes (6, 7).

This paper addresses the following research questions: How can Gen3's architecture be enhanced to support temporal visualization of clinical trial data while maintaining FAIR principles? What technical implementations are required to enable real-time monitoring of longitudinal outcomes across multiple trials? How can standardized temporal analytics be achieved without compromising data security and access controls?

Our implementation maintains FAIR data principles while addressing the specific technical requirements of longitudinal clinical research (8). We demonstrate this framework's effectiveness through its deployment in NIH HEAL Initiative clinical trial networks studying chronic pain and substance use disorders (3). though the approach is broadly applicable across clinical domains (4, 9–11).

## Methods

### System architecture overview

The adaptation of Gen3 for clinical trial applications required significant architectural enhancements to support temporal data visualization and analysis. The core enhancement involved developing a cloud-agnostic implementation that freed the platform from vendor-specific constraints (5). This platform independence proved crucial for research networks that operate across multiple institutions with varying infrastructure requirements. The successful transition between cloud providers demonstrated the feasibility of cross-cloud deployment while maintaining full functionality.

### Cloud-native infrastructure implementation

The implementation leverages comprehensive container orchestration through Kubernetes, with robust node pool configurations enabling automated scaling based on resource utilization metrics. The containerized environment is secured through NeuVector's zero-trust security model (12), providing Layer 7 firewall capabilities and continuous vulnerability scanning (13). This security framework integrates seamlessly with OAuth2 authentication patterns (14), ensuring consistent access controls across all microservices.

### Temporal visualization framework

The integration of temporal visualization capabilities marked another crucial advancement. By incorporating Kibana-based analytics, the platform gained the ability to track longitudinal outcomes effectively. The visualization framework supports interactive filtering and temporal comparisons, enabling researchers to examine treatment effects across multiple timepoints and subgroups (15).

Our implementation integrates Kibana with ElasticSearch indices (16), enabling researchers to examine longitudinal patterns through both predefined and custom dashboard configurations. This implementation includes automated ETL pipelines that maintain data synchronization between PostgreSQL databases (17) and ElasticSearch indices, ensuring near real-time availability of temporal analytics (16, 18, 19).

### Security architecture

**Figure 1:**
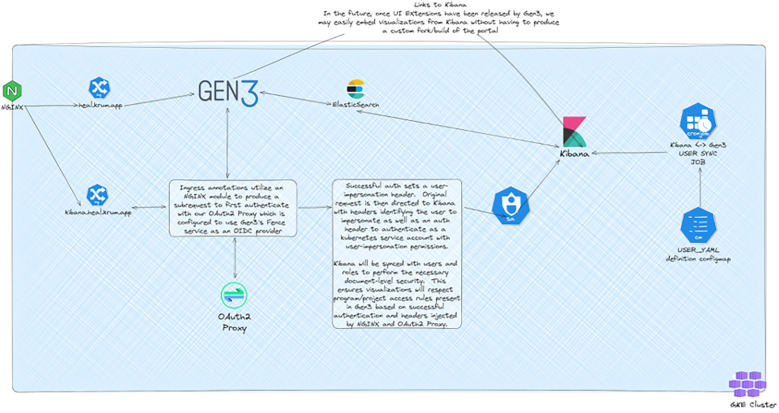
Gen3 authentication and user synchronization architecture. Architectural diagram showing the integration between Gen3, Kibana, and authentication services. The system utilizes NGINX modules for OAuth2 authentication and maintains user synchronization between Gen3 and Kibana to ensure consistent access controls across services. Created using excalidraw, https://excalidraw.com/.

Security enhancements formed a critical component of the adaptation (5). The implementation of NeuVector provided zero-trust container security, crucial for protecting sensitive clinical trial data (20). The security architecture implements comprehensive authentication and user management, specifically OAuth2 for external service authentication and includes comprehensive backup strategies across services, as illustrated in Figure 1 (14). The security model maintains compliance with regulatory requirements while enabling appropriate data sharing and collaboration across research sites.

### ETL pipeline development

**Figure 2:**
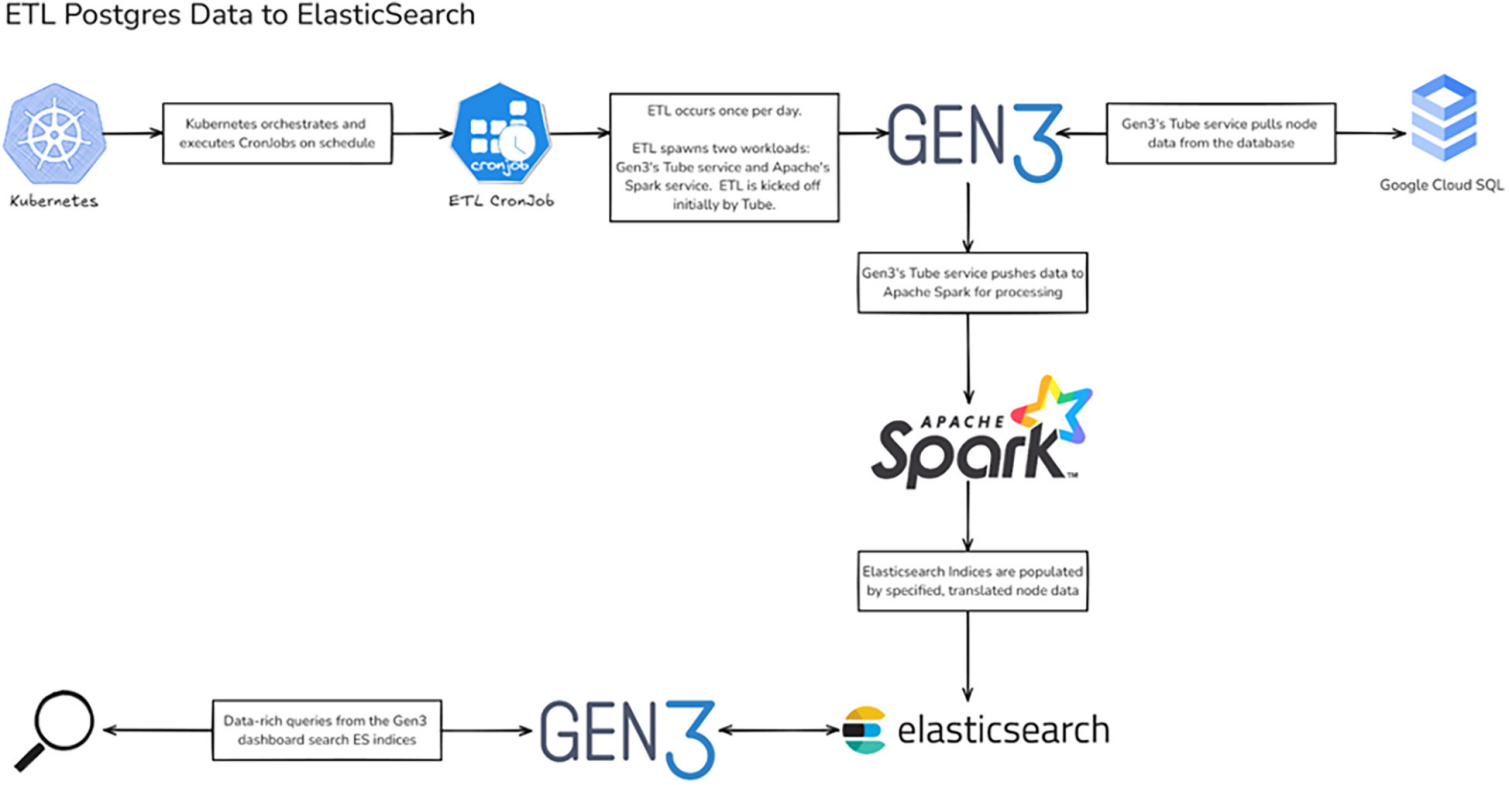
ETL pipeline architecture for Gen3 clinical trial data. Workflow diagram illustrating the ETL process from PostgreSQL to Elasticsearch. The pipeline leverages Kubernetes for orchestration, with Gen3's Tube service coordinating data extraction and Apache Spark handling transformation before loading into Elasticsearch indices. Created using excalidraw, https://excalidraw.com/.

Data harmonization capabilities represent another significant enhancement. The platform now includes automated ETL pipelines for standardizing data across different trials and sites. To ensure consistent data processing and standardization, we developed an automated ETL pipeline architecture with specific adaptations for temporal clinical trial data (21). Our implementation, shown in Figure 2, extends their approach by incorporating clinical trial-specific data validation and temporal relationship preservation. The pipeline includes automated ETL processes that maintain data synchronization between PostgreSQL databases (17) and ElasticSearch indices, ensuring near real-time availability of temporal analytics (16, 18, 19). The enhanced data dictionary management system enables flexible adaptation to different clinical trial protocols while maintaining standardized data structures.

The enhanced data dictionary management system enables flexible adaptation to different clinical trial protocols while maintaining standardized data structures. Validation focused on ensuring accurate representation of clinical trial trajectories and proper handling of temporal data harmonization across diverse measurement types and collection schedules.

## Results

### Temporal analytics validation

The enhanced Gen3 platform's temporal analytics capabilities required specific validation approaches to ensure reliability for clinical trial time series analysis. The integration of Kibana-based visualizations introduced new requirements for validating both data accuracy and analytical functionality across longitudinal datasets (16).

Validation of the temporal visualization framework demonstrated precise handling of varied time-based data structures, from regular visit schedules to irregular event-based capturing. Testing encompassed multiple temporal granularities, from daily patient-reported outcomes to monthly clinical assessments, while maintaining proper temporal relationships between different measurement types. The system successfully handled common clinical trial challenges including missing timepoints, out-of-window measurements, and protocol deviations.

### ETL pipeline performance

The Elasticsearch ETL pipeline validation confirmed accurate transformation of diverse time-based measurements into standardized formats while preserving temporal precision and measurement context. Testing verified proper data synchronization between PostgreSQL databases and ElasticSearch indices, with successful handling of the data volume and complexity typical of multi-site clinical trials.

### System performance and usability

Performance validation addressed the demands of temporal queries and real-time filtering. The platform maintained responsive performance when generating time-based visualizations across large datasets, with successful testing of concurrent users performing temporal analyses. Dashboard refresh rates remained within acceptable limits even when applying complex temporal filters and aggregations, ensuring practical utility for trial monitoring and interim analyses.

**Figure 3:**
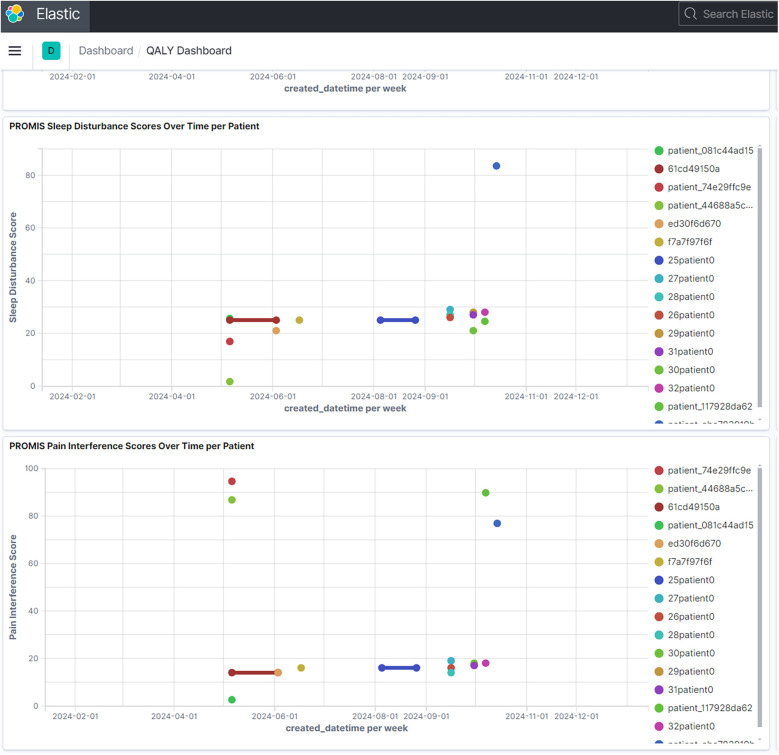
PROMIS patient-reported outcomes dashboard. Example of temporal visualization capabilities showing longitudinal patient-reported outcomes. Multiple measures including sleep disturbance, social activity, pain interference, and pain intensity are tracked over time, demonstrating the platform's ability to handle diverse temporal data streams. (Reproduced with permission from Wake Forest University, https://impowrgen3.wakehealth.edu/login).

**Figure 4:**
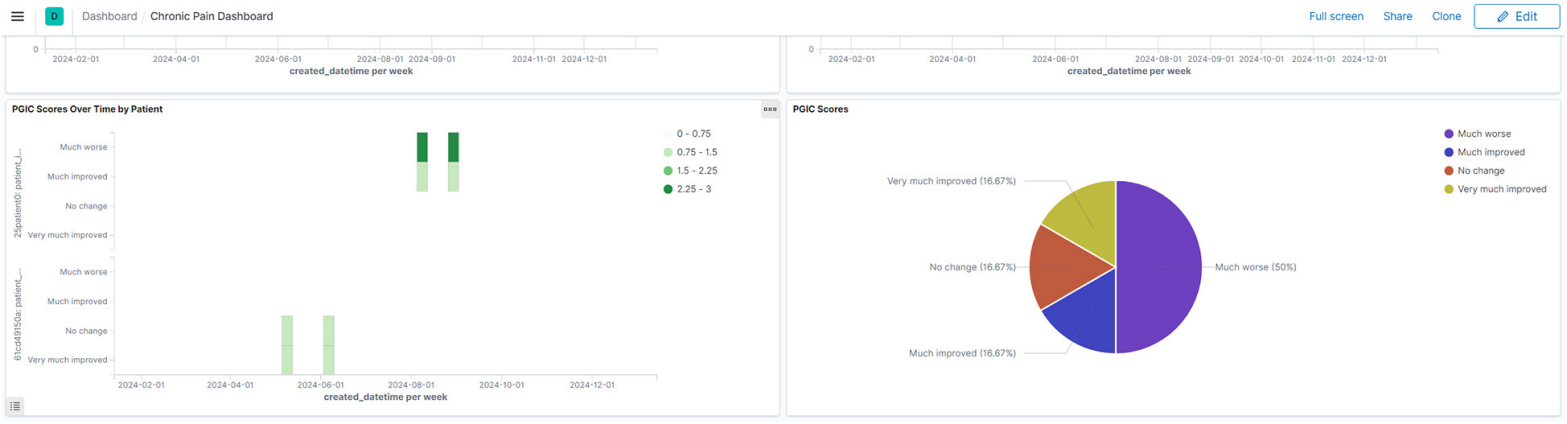
Chronic pain clinical trial monitoring dashboard. Interactive dashboard showing aggregated trial outcomes including PGIC scores and GAD-2 anxiety measures. The visualization demonstrates both temporal trends and distribution patterns, enabling real-time monitoring of trial progress and patient outcomes. (Reproduced with permission from Wake Forest University, https://impowrgen3.wakehealth.edu/login).

The platform's temporal visualization capabilities are demonstrated through a patient-reported outcomes (PROMIS) dashboard (Figure 3), which enables tracking of multiple outcome measures over time. Real-time trial monitoring capabilities are exemplified in the chronic pain clinical trial dashboard (Figure 4), which presents both temporal trends and distribution patterns of key outcome measures.

The platform's automated testing framework incorporates containerized test suites that validate both data integrity and temporal visualization accuracy. Performance validation demonstrated sustained responsiveness under concurrent user loads, with dashboard refresh rates remaining under acceptable thresholds even when applying complex temporal filters across large datasets (22).

## Discussion

The integration of temporal analytics capabilities into the Gen3 data commons platform addresses a fundamental need in clinical research: the ability to understand and analyze treatment effects over time while maintaining the security and standardization benefits of a data commons architecture. Research teams can now track critical outcome measures across multiple timepoints without requiring specialized informatics support.

### Technical contributions and enhancements

The integration of Kibana into the Gen3 stack significantly enhanced visualization capabilities beyond native architecture limitations. Our specific modifications to existing frameworks include: adaptation of ElasticSearch indexing for clinical trial temporal data structures, development of OAuth2 integration patterns for multi-service authentication, and implementation of automated ETL processes specifically designed for patient-reported outcome measures.

### Broader research implications

The standardized approach to temporal visualization enables cross-trial comparisons and meta-analyses, fostering deeper understanding of treatment trajectories across different patient populations and interventions. In the NIH HEAL Initiative networks, researchers can now visualize patterns of patient-reported outcomes across multiple trials (Figures 3,4), leading to insights about treatment effectiveness that were previously difficult to obtain.

These enhancements align with evolving requirements for clinical trial transparency while maintaining appropriate privacy protections. The platform eliminates the need for specialized informatics support through Jupyter notebooks (9), democratizing data access and facilitating independent exploration of temporal patterns, evaluation of potential secondary analyses, and development of new research hypotheses (23).

The enhanced platform demonstrates particular utility in multi-site clinical trials, where real-time monitoring of longitudinal outcomes is essential for trial management. Research coordinators can track enrollment progress and outcome measure completion across sites, while investigators can examine temporal trends in key endpoints as they emerge.

### Future directions

Integration with emerging clinical trial standards and data models presents a key opportunity for expanding interoperability. Development of more sophisticated statistical analysis tools integrated with temporal visualizations represents another promising direction, while maintaining the platform's emphasis on user-friendly interfaces.

The current implementation's cloud-agnostic architecture positions it well to incorporate emerging technologies for improved performance and scalability. Expansion of temporal visualization capabilities to support adaptive trial designs and integration with real-time data streams from wearable devices could extend the platform's utility for modern clinical trial designs.

The growing emphasis on patient-centered research suggests a need for developing interfaces that can effectively communicate temporal patterns to trial participants while maintaining appropriate data protections and scientific rigor.

### Limitations

Several limitations should be acknowledged in this work. The technical implementation requires specific infrastructure and expertise that may not be readily available at all research institutions. The platform's dependency on the Kubernetes, Elasticsearch, and Kibana technology stack creates potential vendor lock-in despite efforts to maintain cloud-agnostic deployment.

From a clinical implementation perspective, the system requires training for clinical staff and may introduce workflow changes that could temporarily disrupt established data collection processes. The data migration process from existing clinical trial management systems presents potential challenges for ongoing studies.

The generalizability of this implementation may be limited by its specific design for NIH HEAL Initiative requirements. While the approach is broadly applicable, adaptation to other clinical trial contexts may require significant customization. The resource requirements for full implementation, including technical expertise and infrastructure costs, may limit adoption in resource-constrained research environments.

Finally, while the platform demonstrates improved temporal visualization capabilities, long-term usability studies and comprehensive user satisfaction assessments were not conducted as part of this implementation project.

## Conclusion

The enhancement of the Gen3 data commons platform to support temporal analytics and dynamic data visualizations represents a crucial advancement for clinical trial infrastructure. Through the implementation of cloud-native architecture and integrated visualization capabilities, Gen3 now provides a framework that addresses fundamental needs in clinical trial data sharing and analysis while adhering to FAIR principles.

The specific technical contributions include: successful integration of temporal visualization tools with existing data commons architecture, development of automated ETL pipelines for clinical trial data harmonization, implementation of security frameworks suitable for multi-site clinical research, and creation of user-friendly interfaces that eliminate the dependency on specialized informatics support. These enhancements demonstrate measurable improvements in data accessibility and analytical capabilities for clinical trial networks.

The success of this implementation provides important lessons for the broader research community. It demonstrates that established data commons platforms can be effectively adapted for specialized research domains without compromising core data sharing capabilities. The cloud-agnostic approach ensures platform sustainability and broad applicability, while thoughtful integration of visualization tools enhances the utility of shared research data.

As clinical trials become increasingly complex and data-intensive, sophisticated yet accessible data commons platforms will continue to be essential. The enhancements described here provide a foundation for future developments in clinical trial data sharing infrastructure. By enabling robust temporal analytics while maintaining security and standardization, this approach advances the goal of making clinical trial data more findable, accessible, interoperable, and reusable for the broader research community.

## Data Availability

The original contributions presented in the study are included in the article/Supplementary Material, further inquiries can be directed to the corresponding author.
